# Feasibility of in-vivo 4D prompt-gamma treatment verification in proton therapy for pancreatic cancer

**DOI:** 10.1016/j.phro.2026.101027

**Published:** 2026-06-20

**Authors:** Jonathan Berthold, Lena Nenoff, Stefanie Bertschi, Julia Thiele, Fabian Lohaus, Guillaume Janssens, Julien Smeets, Kristin Stützer, Christian Richter

**Affiliations:** a OncoRay – National Center for Radiation Research in Oncology, Faculty of Medicine and University Hospital Carl Gustav Carus, TUD Dresden University of Technology, Helmholtz-Zentrum Dresden-Rossendorf, Dresden, Germany; b Center for Advanced Systems Understanding, CASUS, Görlitz, Germany; c Helmholtz-Zentrum Dresden-Rossendorf, CASUS - Center for Advanced Systems Understanding, Dresden, Germany; d Helmholtz-Zentrum Dresden-Rossendorf, Institute of Radiooncology – OncoRay, Dresden, Germany; e Faculty of Medicine Carl Gustav Carus, TUD Dresden University of Technology, Dresden, Germany; f Department of Radiotherapy and Radiation Oncology, Faculty of Medicine and University Hospital Carl Gustav Carus, TUD Dresden University of Technology, Dresden, Germany; g Ion Beam Applications SA, Louvain-la-Neuve, Belgium; h National Center for Tumor Diseases (NCT), NCT/UCC Dresden, a partnership between DKFZ, Faculty of Medicine and University Hospital Carl Gustav Carus, TUD Dresden University of Technology, and Helmholtz-Zentrum Dresden-Rossendorf (HZDR), Germany; i German Cancer Consortium (DKTK), Partner Site Dresden, and German Cancer Research Center (DKFZ), Heidelberg, Germany

**Keywords:** proton therapy, treatment verification, range verification, pancreatic cancer, prompt-gamma, prompt gamma imaging

## Abstract

**Background and purpose:**

Prompt-gamma imaging (PGI) can detect deviations between planned and delivered proton spots. To date, PGI has been limited to body sites not influenced by regular motion or substantial intrafraction anatomical changes that can affect the proton range during treatment delivery. This study investigates the feasibility of time-resolved (4D) PGI treatment verification with a PGI slit camera, incorporating breathing motion in a first patient application.

**Material and methods:**

Synchronously acquired beam delivery (log files), breathing phase and PGI data are evaluated in 3D and 4D for four fractions with control CTs of a pancreatic cancer patient.

**Results:**

The first 4D PGI workflow was developed. The median range shifts between control CT-based simulation and PGI measurements were < 2.3 mm. Planning-CT-based interfraction PGI evaluations showed good agreement between planned and delivered spots for early fractions (median shift < 2.1 mm), but larger deviations (median shift ∼ 6 mm) for later fractions, related to progressive patient weight loss visible on the control CTs. Differences between 3D and 4D PGI were small, due to breathing suppression applied as part of the standard clinical protocol.

**Conclusions:**

A 4D PGI workflow has been successfully developed and tested on a pancreatic-cancer patient, demonstrating the feasibility of PGI-based treatment verification for patients with tumors affected by respiratory motion, even though the proof-of-concept measurements were done with breathing suppression.

## Highlights

• A 4D prompt-gamma-imaging treatment verification workflow was developed.
• Measurements were compared to motion-aware 4D prompt gamma simulations.
• 4D treatment verification was applied in a clinical proof-of-concept study.
• This has potential to enable treatment verification for patients with moving tumors.

## Graphical abstract

**Figure:**
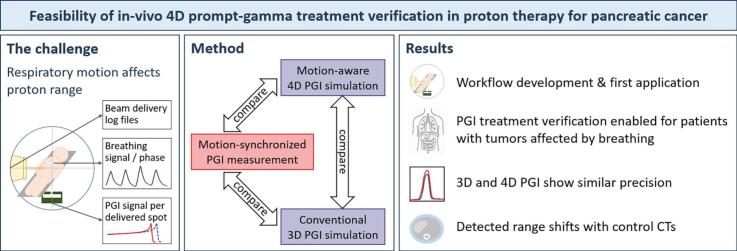

## 1 Introduction

During the proton therapy treatment course, the patient's anatomy can change, leading to deviations between the planned and delivered dose [1], [2]. Treatment verification with prompt-gamma imaging (PGI) can be applied to detect anatomical changes influencing the proton range [3]. A PGI slit camera prototype [4] has been thoroughly studied in anthropomorphic phantoms [5], [6], [7] and is under clinical investigation since 2015 [8], [9], [10], resulting in several systematic retrospective clinical investigations [11], [12], [13]. PGI has enabled the independent validation of our CT-based range prediction in patients [11] and its potential application for a margin reduction in prostate cancer treatments was quantified [12]. Furthermore, the capability of PGI to detect anatomical changes during prostate cancer treatment was evaluated [13].

Thus far, spot-wise – and therefore time-resolved - PGI measurements have been compared to reference PGI profiles simulated on static three-dimensional (3D) computed tomography (CT) datasets, that are not affected by regular motion. However, breathing can influence the proton range [14], and a previous simulation study for a lung-cancer patient has shown that PGI signals vary with breathing motion [15]. The time-resolved (4D) range verification of pencil-beam scanning (PBS) treatments could be evaluated by comparing the measurements to motion-aware 4D simulations that consider the anatomy of the respective breathing phases during delivery. This may lead to a more accurate detection of actual anatomical changes than a 3D evaluation allows, and extend the benefits of PGI treatment verification to patients with tumors affected by respiratory motion. In the context of online adaptive proton therapy, PGI could serve as an independent safety net by detecting treatment deviations without the need for additional imaging dose. PGI could also help to improve treatment safety and reduce treatment margins for patients with moving tumors in the future, after careful clinical studies similar to those in static cases for prostate cancer patients [12]. To realize 4D PGI, spot-wise PGI measurements should be compared to a 4D reference PGI simulation that considers the anatomy of the corresponding breathing phase during which each spot was delivered, rather than to a 3D reference PGI simulation based on the average CT.

In this proof-of-concept study, we demonstrated the feasibility of 4D PGI treatment verification for a pancreatic cancer patient based on the synchronized measurements of breathing phase, beam delivery timing, and PGI information.

## 2 Materials and methods

### 2.1 Patient data

A 63-year-old female patient with a histologically confirmed, inoperable pancreatic cancer (T3N0M0) recurrence two years after initial treatment (resection and chemotherapy) was referred to proton therapy. The treatment plan was designed to deliver 66 Gy (RBE) with a relative biological effectiveness of 1.1^3^ in 30 fractions to the high-risk clinical target volume (CTV) and 51 Gy (RBE) to the low-risk CTV. As is standard practice for pancreatic cancer patients in our clinical treatments, a breathing suppression belt was applied, limiting the patient's remaining breathing motion to <3 mm for this patient. For four fractions (11, 15, 23 and 25), the delivery of one of three beams (275°, lateral direction) was monitored with PGI (PRIMA: DRKS00009224, ethics approval: EK181042015) and a control CT (cCT) was acquired. For fractions 15 and 25, the residual breathing motion was monitored in parallel to the beam delivery and PGI acquisition. 4D cCTs were also acquired on these days. The rigid registrations of all cCTs with the planning CT (pCT) were checked by an experienced RTT. Table 1 provides an overview of the CTs and breathing monitoring for the PGI-monitored fractions.

**Table 1:** Overview of the imaging and breathing monitoring for the fractions monitored with PGI.

Fraction	11	15	23	25
cCT	3D (in-room)	4D + average (off-room)	4D + average (off-room)	4D + average (off-room)
Breathing monitoring	No	Yes	No	Yes
PGI evaluation	3D	3D + 4D	3D	3D + 4D

### 2.2 PGI slit camera and PGI data processing

The PGI prototype used in this study [4] consisted of a knife-edge slit collimator (effective slit width of 11 mm for 4.4 MeV photons) and two rows of 20 Lutetium-Yttrium Oxyorthosilicate (LYSO) crystal slabs, each with a width of 4 mm in beam direction and an energy resolution of ∼5% at 5.1 MeV. It was mounted on a trolley with a floor-based docking system [11] ensuring a fast and reproducible positioning (2σ = 1 mm). The setup of the PGI system was the same for all measurements with the field of view (FOV) focused on the distal fall-off of the treatment field, leading to an overall detection efficiency of 4⨯10^−4^ [5]. Signals from the 13% of spots stopping outside the effective FOV of the PGI camera (±5 cm along the central beam axis) were filtered out during the evaluation.

PGI reference simulations were performed on all average (3D) or 4D CTs using an analytical tool [16], [17]. The simulation included two steps (Supplement A): (1) ray tracing of the proton beam and determination of the prompt gamma radiation emitted by the interaction of the proton beam with the patient geometry provided by a CT dataset, and (2) modeling of the detector response leading to a one-dimensional (1D) PGI profile per spot. For all measured and simulated PGI spot profiles a two-dimensional Gaussian (σ = 7.8 mm) aggregation of PGI profiles from spots within the same energy layer was done in order to reduce the influence of statistical noise on the shift retrieval [7]. The measured PGI profiles were compared to different 3D or 4D simulated reference profiles. The study design also included the comparison of profiles from different PGI simulations, as outlined in the section on study design below. In any profile comparison, spot-wise range shifts between the aligned profiles were retrieved using a 1D least-squares matching with a focus on the distal fall-off [6].

### 2.3 Breathing monitoring and 4D PGI simulation

All 4D CTs were acquired using surface monitoring (Sentinel, C-RAD AB, Uppsala, Sweden). Eight amplitude-sorted breathing phases and an average CT were reconstructed. During the delivery of fractions 15 and 25, the breathing motion signal was detected with a pressure belt system (AZ-733 V, Anzai Medical, Japan). The timing information from the pressure belt system and the beam delivery log files were synchronized, and the spots of the treatment plan were sorted to the corresponding breathing phase. Eight subplans containing the log file-determined spot positions and number of monitor units delivered per spot were generated [18], [19]. These subplans were then used to simulate PGI profiles on the respective breathing phase of the 4D CT (pCT or cCT, respectively). Finally, phase-wise simulated PGI profiles were merged to obtain a motion-aware 4D PGI simulation. In contrast, a 3D PGI simulation was created using only the average 3D CT and the spot positions from the nominal treatment plan as inputs.

### 2.4 Study design

**Fig. 1:**
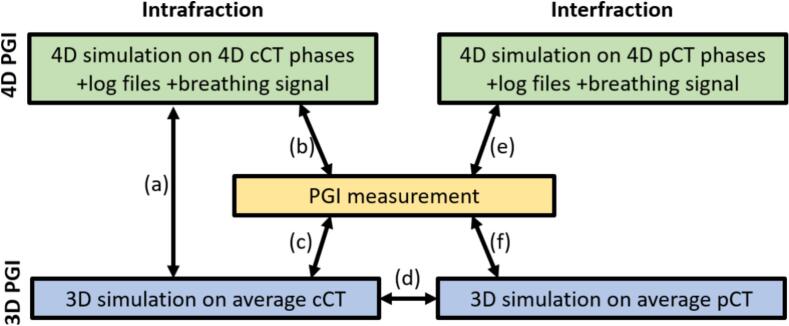
Evaluation scheme between 4D (green) and 3D (blue) prompt-gamma-imaging (PGI) simulations and PGI measurements (yellow). The evaluation is separated between intrafraction evaluation (left) using simulations on the control CT (cCT) and the interfraction evaluation (right) using simulations on the planning CT (pCT). (For interpretation of the references to colour in this figure legend, the reader is referred to the web version of this article.)

We conducted six evaluations (Fig. 1) to determine range shifts between either two PGI simulations or a PGI measurement and a simulation. These evaluations were separated into interfraction and intrafraction evaluations. Intrafraction evaluations were comparisons with reference simulations on the cCT, and interfraction evaluations were comparisons with reference simulations on the pCT. Although we were aware that PGI measurements are always affected by potential intrafraction changes that occur during data acquisition, for simplicity, we refer to all PGI comparisons with 3D or 4D simulations on the pCT as “interfraction“evaluations. Range shift histograms and medians were reported for all evaluations.

First, the daily ground truth anatomy, given by the cCTs, was considered (intrafraction evaluations): For fractions 15 and 25, 4D PGI simulations were compared to (a) 3D PGI simulations to check the influence of the breathing motion, and (b) the measured PGI data to investigate the additional effect of measurement uncertainties. Additionally, (c) PGI measurements from all fractions were also compared to the standard 3D PGI simulations.

Second, PGI treatment verification is typically performed by comparing PGI signals of the respective fraction to simulations on the pCT anatomy (interfraction evaluation). The influence of the interfraction changes was determined by (d) comparing 3D PGI simulations on the pCT to those on the cCTs for all fractions. Furthermore, range shifts between the PGI measurements and (e) the 4D PGI simulations for fractions 15 and 25, and (f) the standard 3D PGI simulations for all fractions, were determined to compare the performance of both methods under typical clinical conditions.

## 3 Results

### 3.1 Intrafraction evaluation with the control CTs

**Fig. 2:**
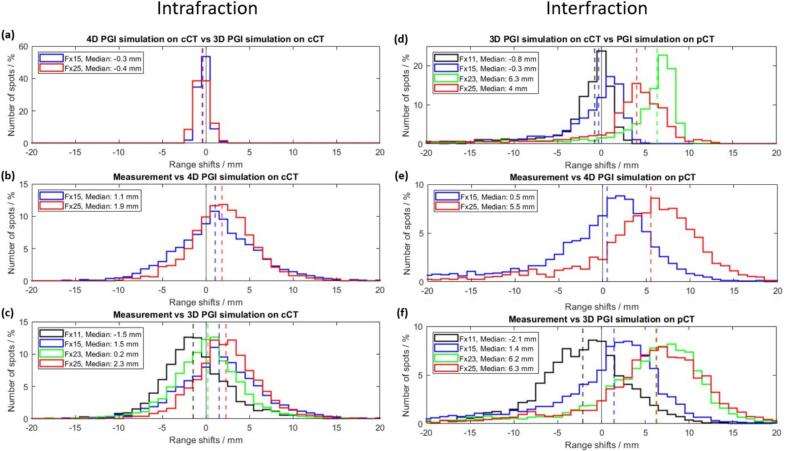
Range shift histograms between a) 4D and 3D PGI simulations of PGI profiles on the cCTs, b) PGI measurements and 4D PGI simulations on cCT, c) PGI measurements and 3D PGI simulation on cCT, d) 3D PGI simulations on cCT and pCT, e) PGI measurements and 4D PGI simulation on pCT and f) PGI measurements and 3D PGI simulation on pCT. (Abbreviations: pCT – planning CT, cCT – control CT, Fx – Fraction)

The intrafraction results were summarized in Figs. 2a–c. The motion influence (3D versus 4D simulation, Fig. 2a) resulted in median range shifts < 1 mm for both fractions, indicating that no relevant differences were expected between 3D and 4D PGI evaluations for this patient. The comparison of both 3D and 4D simulations to the PGI measurements (Figs. 2b–c) showed broader distributions but comparable results, with median range shifts between −1.5 mm and 2.3 mm. Notably, for the two fractions with both 3D and 4D PGI evaluations available, the measured median range shifts were closer to zero by ∼ 0.4 mm for the 4D PGI simulation (1.1 mm and 1.9 mm, respectively; Fig. 2b) than for the 3D PGI simulation (1.5 mm and 2.3 mm, respectively; Fig. 2c). This suggests a slightly improved agreement between measured and simulated proton range when respiratory motion is explicitly considered. However, meaningful results could also be achieved from 3D PGI evaluations for this specific patient.

### 3.2 Interfraction evaluation with the planning CT

The interfraction results were summarized in Figs. 2d–f. The influence of the interfraction changes (comparison between 3D PGI simulations on the cCT and pCT, Fig. 2d) was negligible at the start and mid of the treatment course (fractions 11 and 15), with a median shift ≤1 mm. However, towards the end of treatment (fractions 23 and 25), median range shifts were 6.3 mm and 4.0 mm, indicating relevant interfraction anatomical changes. These findings were consistent when comparing real-world PGI measurements to the pCT-based 3D or 4D PGI simulations, as in a typical clinical PGI application (Fig. 2e and f): For fractions 11 and 15, the absolute median shifts were ≤ 2.1 mm, similar to the comparison with intrafraction cCT-based PGI simulations. For fractions 23 and 25, larger median shifts of 6.2 mm and 6.3 mm, respectively, were detected when comparing to 3D PGI simulations (Fig. 2f). Comparing the measurements to 4D PGI simulations confirmed these results, with median range shifts of 0.5 mm in fraction 15 and 5.5 mm in fraction 25 (Fig. 2e).

**Fig. 3:**
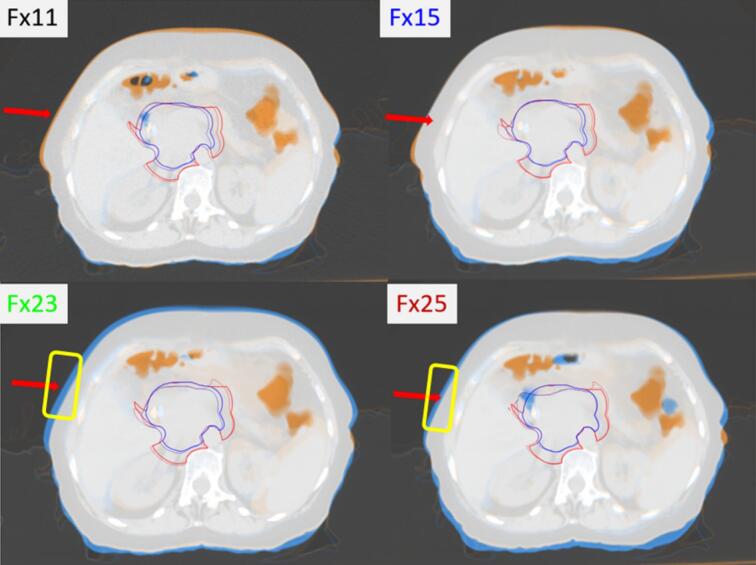
Difference between planning CT (sky blue) and control CTs (orange) for each monitored fraction. The patient's weight loss was visible towards the end of treatment. High-risk and low-risk CTV are shown in dark blue and red, respectively. The direction of the monitored beam is indicated by the red arrow. Yellow boxes highlight the area of the patient surface shift. (For interpretation of the references to colour in this figure legend, the reader is referred to the web version of this article.)

The patient's weight loss of 5 kg between the pCT and the cCTs at the end of treatment was also visible in a shift of the patient surface (Fig. 3). This shift in the patient surface resulted in an increased proton penetration, leading to an overshoot of 4–6 mm in fractions 23 and 25, as detected by the PGI evaluation. Nevertheless, the CTV coverage and organ doses remained acceptable throughout the treatment.

## 4 Discussion

We clinically applied PGI-based treatment verification for the first time to an abdominal target volume. Since this body region is subject to intrafraction respiratory motion, we established a 4D PGI evaluation based on the synchronous acquisition of breathing motion, beam delivery, and PGI data. The correct performance of the new evaluation approach, including the generation of a 4D PGI simulation, was confirmed.

The comparison of the 4D and 3D PGI simulations demonstrated only a minor influence of respiratory motion on proton range for the patient studied. This likely reflects the effect of the used abdominal compression and the limited anatomical variation along the beam path, findings that were further supported by spot-wise integrated depth dose calculations (Supplement 2). Importantly, the successful implementation of a technically realistic 4D PGI workflow demonstrated the feasibility of extending PGI treatment monitoring to patients with moving targets. The measured range shifts were comparable to those obtained with conventional 3D PGI evaluation and appeared to track gradual changes throughout the treatment course, most likely attributable to patient weight loss. These results suggest that the established benefits of PGI, including independent verification of proton range, detection of anatomical changes without additional imaging dose, and support for margin reduction strategies, may also be achievable in selected patients with respiratory motion. Further studies are needed to evaluate the clinical value across a broader range of disease sites and motion scenarios.

For a clinical evaluation, it is important to categorize range shifts as either clinically relevant or non-relevant. A first proposal to define a relevant anatomical change in the context of range verification was made for prostate cancer treatments [13]. The proposal suggested that if ≥1.5% of the spot weights per treatment field are affected by a range shift of at least 5 mm, the underlying anatomical change is considered “relevant“. This does not mean that the current clinical goals for that treatment are automatically failed. Instead, this definition already accounts for potential margin reduction in online adaptive proton therapy and is more sensitive to smaller changes than in current clinical practice. However, the development and validation of a PGI-based classification model for pancreatic cancer patients would require a substantially larger patient cohort and was outside the scope of this proof-of-concept study.

Due to time constraints in the treatment room and the desire to minimize the patient's time on the treatment couch, breathing motion was only monitored for two fractions in this study. Despite the small number of fractions, we achieved the goal of this proof-of-concept study: the developed 4D PGI workflow is feasible, and the obtained data can be successfully processed. Additionally, this study relied on off-room CTs for all 4D CTs, which required patient repositioning between the PGI measurement during treatment delivery and the CT acquisition. Only a single in-room (3D) CT was available for comparison. Nevertheless, the range shift histograms with absolute median range shifts of less than 2.3 mm, obtained from comparisons between measurements and cCT-based simulations (Fig. 2b and c), were consistent with findings from a previous prostate-cancer study that used pre-delivery in-room CTs. In this study, a precision of 3 mm (2σ) for the mean PGI range shift was reported, with the dominant sources of uncertainty arising from the camera positioning and patient setup [12]. Thus, we found no evidence for a systematic bias from the use of off-room rather than in-room cCT for PGI evaluations in this study.

We are currently implementing a fast workflow for an online evaluation in an interventional clinical study of prostate cancer treatments. The reference simulation takes several hours and can be performed before the fraction delivery. A full 3D PGI evaluation, including a binary classification for a clinically relevant deviation, can be processed within ∼5 min after field delivery. For the retrospective study presented here, all simulations were performed after the treatment and took several hours. Rather than sorting the delivered spots to the CT phases and then performing the reference simulation on the respective 4D CT phase after each fraction, PGI simulations with all spots could be precalculated for each 4D CT phase after plan approval. After fraction delivery, the respective pre-simulated profile could be selected from the correct breathing phase for each spot and compared to the measurements. We estimate that the additional processing time for 4D PGI-related tasks will increase the overall processing time by only a few minutes compared to the 3D PGI evaluations. Therefore, an online application comparable to our 3D PGI interventional study also seems feasible for 4D-PGI.

In principle, with a future implementation of online readout, PGI data could be evaluated instantly after the delivery of each individual spot. This would allow to further exploit one of PGI's main advantages over other in-vivo range verification methods: the instantaneous gamma emission occurring within picoseconds after the nuclear interaction enables PGI signal detection in real-time. This is also the key advantage of PGI over treatment verification based on positron emission tomography (PET), for which the impact of organ motion on treatment verification has been investigated previously [20], [21], [22], [23], [24]. Most PET isotopes generated during treatment have a half-life of several seconds to minutes and are subject to biological washout. This complicates conclusions about the origin of treatment deviations. However, prompt gamma-based verification methods require higher detector throughput and signal processing, which is one of the main reasons for their slow advancement into clinical use.

Our results demonstrated the successful development and general feasibility of a 4D PGI workflow in a clinical setting. However, during the first clinical demonstration, the workflow was only applied to one patient who was treated with breathing suppression. This was necessary, since the initial proof-of-concept study was designed to not interfere with the treatment decisions. The primary objective is the safe treatment of the patient, which requires breathing suppression in the abdominal region at our institute. In the long term, combining 4D PGI with a fast, automatic online feedback loop could potentially allow the treatment of these patients in free breathing. This could make proton therapy more comfortable. However, this requires a systematic clinical study with real-time or daily 4D PGI evaluation, as well as a separate ethics vote, neither of which was intended in this study.

The 4D CTs were acquired using a surface-guiding system, while during the treatment delivery the breathing was monitored using a pressure belt system. The agreement between both systems was confirmed with phantom experiments during clinical 4D CT commissioning.

This first clinical investigation of 4D PGI treatment verification showed no relevant benefits over 3D PGI in this single patient. However, this finding cannot be generalized, and more studies are needed to evaluate the effectiveness of 4D PGI in treating not only abdominal but also thoracic tumors. Additionally, possible dependencies on anatomical areas (i.e. influence of sharp density gradients in lung), breathing amplitude, and frequency should be investigated.

In conclusion, the general feasibility of 4D PGI was demonstrated in a pancreatic cancer treatment. In this case, no relevant difference was found between 3D and 4D PGI. However, we detected relevant interfraction changes and confirm them with cCT images. While the results of this proof-of-concept case study are not generalizable, they mark the first step in the clinical implementation and application of 4D PGI.

## Declaration of generative AI use

The authors used suggestions from DeepLWrite (Free) during the revision to improve the clarity of specific sentences and paragraphs.

## CRediT authorship contribution statement

**Jonathan Berthold:** Writing – review & editing, Writing – original draft, Visualization, Validation, Software, Methodology, Investigation, Formal analysis, Data curation, Conceptualization. **Lena Nenoff:** Writing – review & editing, Writing – original draft, Visualization, Validation, Supervision, Software, Methodology, Investigation, Formal analysis, Data curation, Conceptualization. **Stefanie Bertschi:** Writing – review & editing, Software, Data curation. **Julia Thiele:** Writing – review & editing, Data curation. **Fabian Lohaus:** Writing – review & editing, Investigation. **Guillaume Janssens:** Writing – review & editing, Validation, Software. **Julien Smeets:** Writing – review & editing, Validation. **Kristin Stützer:** Writing – review & editing, Supervision, Methodology, Data curation, Conceptualization. **Christian Richter:** Writing – review & editing, Supervision, Funding acquisition, Data curation.

## Declaration of competing interest

The authors declare the following financial interests/personal relationships which may be considered as potential competing interests: OncoRay has institutional research agreements with Ion Beam Applications S.A. (IBA). G. Janssens and S. Smeets are employees of IBA. OncoRay also has an institutional research agreement with Siemens Healthineers in the field of CT imaging for particle therapy. For the present study, the authors received no financial support, neither for the design of the study or the selection of the materials used, nor for the collection, analysis and interpretation of the data nor for the preparation of the publication. The authors report no conflict of interest.

## Footnotes

3 Following our clinical standard and the ICRU78.
