# Supplementary material for: Feasibility of in-vivo 4D prompt-gamma treatment verification in proton therapy for pancreatic cancer
Source: Phys Imaging Radiat Oncol. 2026 Jun 20;40:101027. doi: 10.1016/j.phro.2026.101027 (PMC13355200; doi:10.1016/j.phro.2026.101027)
Supplement: Supplementary file 1 — Supplementary material [file mmc1.pdf]

Supplement A: Room-fixed PGI slit camera and PGI simulation

The PGI slit camera used in this study was mounted on a second-generation trolley with the orientation of the collimator plane being horizontally fixed for any field delivery (room-fixed camera). This design limitation of the PGI system (which is not a general limitation of PGI) was chosen to ensure a fast and reproducible setup of the PGI system during patient treatments (Berthold et al., 2018). The measured PGI signals were then compared to a corresponding PGI simulation (Sterpin et al., 2015), which was performed in two steps:

(1) At first, a curved raytracing across the lateral spread of the beam (3σ) was performed with 45 equidistant raytraces and the emitted prompt gamma radiation of the proton beam within the patient CT scan was determined from pre-calculated data. For this step, we transformed the single-energy CTs (SECT) to stopping-power ratio (SPR) maps using an adapted Hounsfield unit look-up table (HLUT) (Wohlfahrt et al., 2020). Subsequently, the voxel-wise SPR information was then converted to material and density according to the MATA (MATerial Assignment) approach (Permatasari et al., 2020). As result of the raytracing step, the emission of prompt gamma radiation was scored along the respective spot axis, going through the virtual magnetic deflection point of the beam.

(2) Secondly, the response of the PGI detector is modelled for every treatment spot. In the current version of the simulation software, this was realized as a 1D projection along a beam axis that was parallel to the collimator plane and perpendicular to the slit (Figure S1). That meant for horizontal treatment fields, the small deflection angle by the scanning magnets was neglected. However, for oblique (non-horizontal) field angles, this is not a valid approximation anymore, if the second-generation trolley system with its room-fixed camera setup is used. For this reason, the simulation software was updated: A projection step prior to applying the transfer function of the detector response was introduced (Figure S1). This ensured that the emitted gamma radiation was first correctly projected onto the 1D axis that is parallel to the collimator plane and perpendicular to the slit, as required for the response modelling. To account for remaining differences between simulated and actual camera response due to effects of the detector geometry and neutron background contribution, we applied the geometrical corrections introduced by Petzoldt et al. (2018) as first-order approximation of these effects.

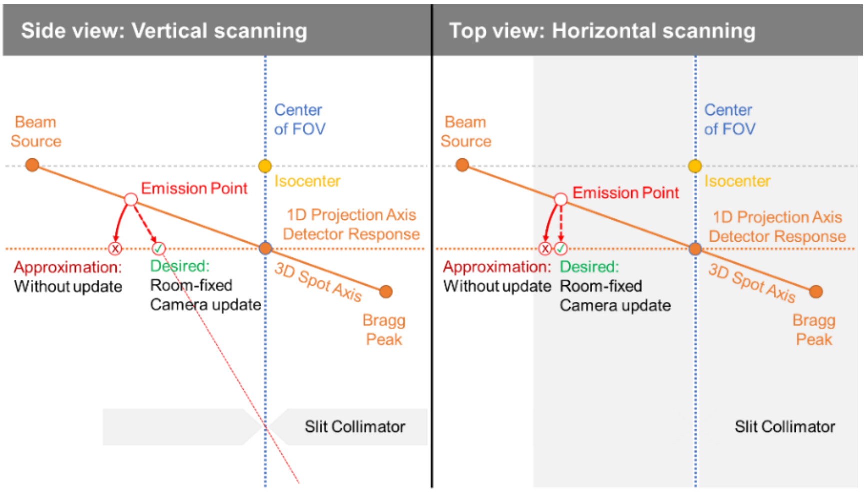

Figure S1: *Update of simulation model by adding the desired projection step onto the horizontal 1D axis used for modelling the detector response when measuring with the trolley system that provides a room-fixed detector setup.*

For non-horizontal treatment fields as for the monitored field in this study (275° instead of 270°), the central axis intersects with the collimator plane of the room-fixed camera, resulting in a compression of the projected PGI signals compared to a parallel alignment. For a deviation of 5° from a horizontal field angle this effect was very small.

References

Berthold J, Khamfongkhruea C, Petzoldt J, Thiele J, Hölscher T, Wohlfahrt P, et al. First-In-Human Validation of CT-Based Proton Range Prediction Using Prompt Gamma Imaging in Prostate Cancer Treatments. Int J Radiat Oncol Biol Phys 2021;111:1033–43. https://doi.org/10.1016/j.ijrobp.2021.06.036

Sterpin, E., Janssens, G., Smeets, J., Stappen, F. Vander, Prieels, D., Priegnitz, M., Perali, I., & Vynckier, S. (2015). Analytical computation of prompt gamma ray emission and detection for proton range verification. Phys Med Biol, 60(12), 4915–4946. https://doi.org/10.1088/0031-9155/60/12/4915

Permatasari, F. F., Eulitz, J., Richter, C., Wohlfahrt, P., & Lühr, A. (2020). Material assignment for proton range prediction in Monte Carlo patient simulations using stopping-power datasets. Phys Med Biol, *65*(18). https://doi.org/10.1088/1361-6560/AB9702

Wohlfahrt, P., Möhler, C., Enghardt, W., Krause, M., Kunath, D., Menkel, S., Troost, E. G. C., Greilich, S., & Richter, C. (2020). Refinement of the Hounsfield look-up table by retrospective application of patient-specific direct proton stopping-power prediction from dual-energy CT. Med Phys. https://doi.org/10.1002/mp.14085

Petzoldt, J., Janssens, G., Nenoff, L., Richter, C., & Smeets, J. (2018). Correction of Geometrical Effects of a Knife-Edge Slit Camera for Prompt Gamma-Based Range Verification in Proton Therapy. Instruments, 2(4), 25. https://doi.org/10.3390/instruments2040025

Supplement B: Correlation of PGI simulation and IDD calculation

Motivation

For an effective treatment verification technique, we need to be able to reliably detect deviations from the expected dose distribution within the patient. PGI is a technique that is able to detect differences in the spot-wise emission profile of prompt gamma radiation, which are usually quantified as a spot-wise range shift along the beam path. A high correlation of the PGI signal with the distal dose fall-off has been proven (Min et al., 2006). However, it is also known that this correlation can be deteriorated by tissue inhomogeneities (Priegnitz et al. 2015, Priegnitz et al.2016). Here, we investigate how well the PGI range shift acts as a surrogate for actual range shifts of integrated depth-dose profiles within the investigated pancreas-cancer patient.

Integrated depth-dose profiles

For the PGI-monitored treatment field (275°) of the pancreas-cancer patient, we retrieved spot-wise integrated depth-dose (IDD) profiles from a Monte Carlo dose calculation with 0.3% uncertainty using RayStation version 2023B on a uniform dose grid of 1 mm^3. As for the PGI evaluations (a) and (d), we retrieved spot-wise IDD range shifts between the respective IDD profiles. The range shifts between IDD profiles on the average CT (3D) and the respective breathing phases of the subplans (4D) were calculated for the 4D control CTs of fractions 15 and 25. The range shifts between IDD profiles on the average planning CT (3D) and control CTs (average/3D) were calculated for fractions 11, 15, 23 and 25.

Correlation of IDD and PGI

The correlation of IDD and non-aggregated PGI range shifts is shown in Figure S2 for the 3D to 4D cCT evaluation containing the intrafractional motion influence and Figure S3 for the 3D pCT to 3D cCT evaluation containing the interfractional motion influence, respectively. The high correlation coefficients between PGI and IDD data of 0.93 for the intrafraction and 0.90 for the interfraction evaluation, combined with the mean spot-wise range shift difference of 0.04 mm and 0.15 mm, respectively, showed that the PGI range shifts agreed well with the IDD range shifts. For the intrafraction evaluation, the spot-wise difference distribution was substantially narrower than in the interfraction evaluation. We related this to the fact that the anatomy of the patient differed more between fractions than within a breathing cycle of the patient, which is also supported by the larger range shifts occurring in the interfraction evaluation. Altogether, this IDD versus PGI comparison reassures that measured PGI range shifts could reliably detect changes in the dose distribution of a pancreas-cancer patient and highlighted the potential of PGI for treatment verification.

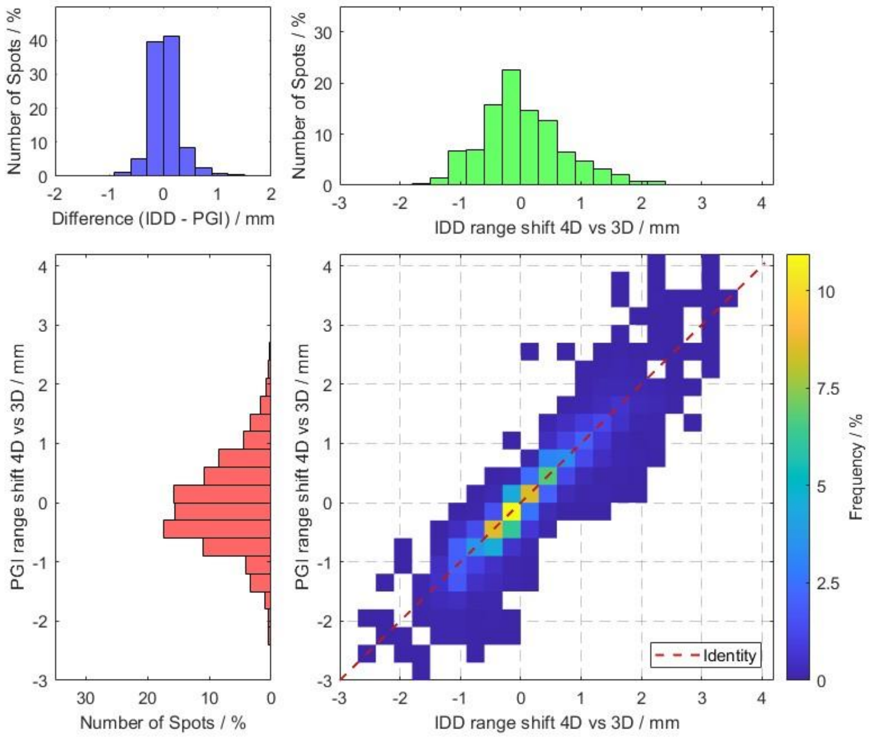

Figure S2: *Intrafraction evaluation: Spot-wise difference, histograms and correlation of IDD and non-aggregated PGI range shifts between 3D and 4D control CT-based simulations. All spots from fractions 15 and 25 are combined.*

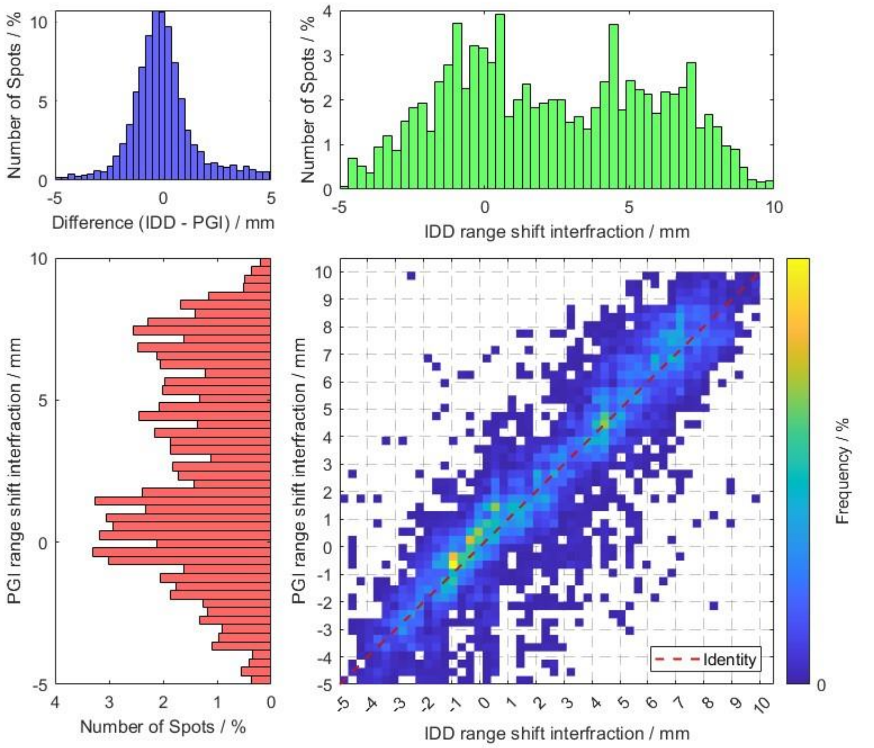

Figure S3: *Interfraction evaluation: Spot-wise difference, histograms and correlation of IDD and non-aggregated PGI range shifts between 3D planning CT- and control CT-based simulations. All spots from fractions 11, 15, 23 and 25 are combined.*

References

Min, C. H., Kim, C. H., Youn, M. Y., & Kim, J. W. (2006). Prompt gamma measurements for locating the dose falloff region in the proton therapy. Applied Physics Letters, 89(18), 183517. https://doi.org/10.1063/1.2378561

Priegnitz, M., Helmbrecht, S., Janssens, G., Perali, I., Smeets, J., Vander Stappen, F., Sterpin, E., & Fiedler, F. (2015). Measurement of prompt gamma profiles in inhomogeneous targets with a knife-edge slit camera during proton irradiation. Phys Med Biol, 60(12), 4849. https://doi.org/10.1088/0031-9155/60/12/4849

Priegnitz, M., Helmbrecht, S., Janssens, G., Perali, I., Smeets, J., Vander Stappen, F., Sterpin, E., & Fiedler, F. (2016). Detection of mixed-range proton pencil beams with a prompt gamma slit camera. Phys Med Biol, 61(2), 855. https://doi.org/10.1088/0031-9155/61/2/855
